# Beyond Screen Time: A Synergistic Approach to a More Comprehensive Assessment of Family Media Exposure During Early Childhood

**DOI:** 10.3389/fpsyg.2020.01283

**Published:** 2020-07-10

**Authors:** Rachel Barr, Heather Kirkorian, Jenny Radesky, Sarah Coyne, Deborah Nichols, Olivia Blanchfield, Sylvia Rusnak, Laura Stockdale, Andy Ribner, Joke Durnez, Mollie Epstein, Mikael Heimann, Felix-Sebastian Koch, Annette Sundqvist, Ulrika Birberg-Thornberg, Carolin Konrad, Michaela Slussareff, Adriana Bus, Francesca Bellagamba, caroline Fitzpatrick

**Affiliations:** ^1^Department of Psychology, Georgetown University, Washington, DC, United States; ^2^Human Development and Family Studies, University of Wisconsin–Madison, Madison, WI, United States; ^3^Department of Pediatrics, University of Michigan, Ann Arbor, MI, United States; ^4^School of Family Life, Brigham Young University, Provo, UT, United States; ^5^Department of Human Development and Family Studies, Purdue University, West Lafayette, IN, United States; ^6^Learning Research and Development Center, University of Pittsburgh, Pittsburgh, PA, United States; ^7^OpenLattice, Inc., Redwood City, CA, United States; ^8^Division of Psychology, Linköping University, Linköping, Sweden; ^9^Department of Clinical Child and Adolescent Psychology, Ruhr-University Bochum, Bochum, Germany; ^10^Institute of Information Studies and Librarianship, Charles University, Prague, Czechia; ^11^School of Communication and Media, University of New York in Prague, Prague, Czechia; ^12^Department of Education, University of Stavanger, Stavanger, Norway; ^13^Department of Dynamic and Clinical Psychology, Sapienza University of Rome, Rome, Italy; ^14^Department of Humanities, Université Sainte-Anne, Nova Scotia, NS, Canada

**Keywords:** joint media engagement, digital media, technoference, early childhood, passive sensing, time use activity data, household usage patterns

## Abstract

Digital media availability has surged over the past decade. Because of a lack of comprehensive measurement tools, this rapid growth in access to digital media is accompanied by a scarcity of research examining the family media context and sociocognitive outcomes. There is also little cross-cultural research in families with young children. Modern media are mobile, interactive, and often short in duration, making them difficult to remember when caregivers respond to surveys about media use. The Comprehensive Assessment of Family Media Exposure (CAFE) Consortium has developed a novel tool to measure household media use through a web-based questionnaire, time-use diary, and passive-sensing app installed on family mobile devices. The goal of developing a comprehensive assessment of family media exposure was to take into account the contextual factors of media use and improve upon the limitations of existing self-report measures, while creating a consistent, scalable, and cost-effective tool. The CAFE tool captures the content and context of early media exposure and addresses the limitations of prior media measurement approaches. Preliminary data collected using this measure have been integrated into a shared visualization platform. In this perspective article, we take a tools-of-the-trade approach (Oakes, 2010) to describe four challenges associated with measuring household media exposure in families with young children: measuring attitudes and practices; capturing content and context; measuring short bursts of mobile device usage; and integrating data to capture the complexity of household media usage. We illustrate how each of these challenges can be addressed with preliminary data collected with the CAFE tool and visualized on our dashboard. We conclude with future directions including plans to test reliability, validity, and generalizability of these measures.

## Introduction

Young children are immersed in the digital world. In the United States, Rideout (2017) used what was widely considered the standard approach to measure media usage, conducting a nationally representative survey of retrospective parent-reported screen time (i.e., the time children are intentionally exposed to screens per day). Rideout reported that, on average, children from birth to 23 months old spend 42 min with screens per day, and 2- to 4-year-olds spend 2 h and 39 min per day. Most of this screen time (72%) is spent viewing video content. However, actual exposure to screen media is likely much higher than traditionally reported given that 42% of parents report the TV is on “always” or “most of the time” in their home, whether anyone is watching or not. The context of media exposure is often underreported as well. For example, 24% of children younger than 2 years often or sometimes use screen media in the hour before bedtime. This rate is twice as high (49%) for 2- to 4-year-olds. Despite the prevalence of screen use immediately before sleep, the impact of this exposure is not well understood. Moreover, the media landscape is rapidly evolving; 98% of all homes in the United States have a mobile device, a number that has steadily increased to saturation levels since 2013 (Rideout, 2017). This pattern is similar across the globe (Pew Research Center, 2019). The evolving media landscape presents many challenges to researchers attempting to assess media exposure and effects in young children. Researchers need new tools to meet these challenges. The purpose of this perspective article is to describe current challenges in measuring media use and introduce state-of-the-art digital media assessment tools.

### Why Do We Care About Media Measurement?

High levels of screen time (duration of intentional screen media exposure) have been associated with a number of developmental outcomes. Many researchers have reported associations between early media exposure and outcomes as wide ranging as sleep (Cheung et al., 2017), obesity (Jackson et al., 2009), antisocial behavior (Zimmerman and Christakis, 2007), attention problems (Christakis et al., 2004), and language delays (Zimmerman et al., 2007). Higher screen time has been identified as a key predictor of poorer outcomes in many nations, including Turkey (Dinleyici et al., 2016), Canada (Madigan et al., 2019), and Hong Kong (Fu et al., 2017) and in a recent series of qualitative studies across seven European countries (Chaudron, 2015). Despite multiple studies reporting negative associations between media use and child outcomes, mixed findings abound. For example, the link between attention and media usage is unclear, with some studies reporting no association (e.g., Acevedo-Polakovich et al., 2006; Foster and Watkins, 2010) and others reporting a positive association, at least for certain types of content (e.g., Friedrich and Stein, 1973). Such mixed findings may be accounted for by factors such as developmental constraints, demographics, environmental characteristics, and media content. Nonetheless, many studies continue to adopt a single, unitary, global estimate of children’s screen time, ignoring the moderating effects of individual-, household-, and media-level characteristics.

Contextual theorists (Vygotsky, 1978; Bronfenbrenner and Morris, 2006) argue that it is imperative to measure the interaction between the individual and the changing contexts within which children develop. Despite widespread debate in both popular and academic circles regarding how traditional and newer forms of digital media influence development, very few studies have examined the confluence of the family social context, digital media use by the parent and child, and early learning and language skills (Troseth et al., 2016). Thus, for a more complete understanding of media use and child development, researchers must investigate not only the duration of media use, but also the developing child within different contexts (e.g., shared use with parents, use during different family routines). However, methods available to collect such contextual knowledge are typically limited. Few studies have included assessments of mobile and interactive media use, particularly among families of very young children. As technology evolves, researchers need to develop measures to complement surveys often focused on screen time. A comprehensive and systematic set of media assessment tools is therefore needed to assess usage in a rapidly changing media landscape.

Furthermore, conclusions are plagued by multiple measurement problems (see Vandewater and Lee, 2009; Barr and Linebarger, 2017 for a review and critique of methods). Observational methods are critical in child- and family-focused research because they reflect the typical behavior of participants in naturalistic settings and because they are capable of chronicling the complex and changing processes that occur daily in young children’s lives. Yet such methods are time-consuming and expensive. Observational methods also require highly trained staff. For these reasons, most studies use imprecise survey methods (e.g., global estimates with only one question asking parents to estimate TV in a “typical” day) to quantify media use (Vandewater and Lee, 2009; Barr and Linebarger, 2017). Such total time estimates ignore content, despite multiple studies that document content as a critical moderator of media effects, as summarized later. Moreover, survey methods vary widely, precluding comparisons across studies. Finally, few studies consider the overall household usage, despite a growing literature on the extent to which media effects are moderated by contextual factors (e.g., parent coviewing and mediation, parents’ own technology use and “technoference,” timing of use such as television viewing immediately before or during sleep, meals, and play).

### The Role of Media Content and Context

For roughly half a century, researchers have documented the critical importance of media content in determining media effects (for reviews, see Fisch, 2004; Anderson and Kirkorian, 2015; Barr and Linebarger, 2017; Lauricella et al., 2017). Relatively less attention has been given to contextual influences, including household characteristics, parental mediation of child media use, and parents’ own media use. Nonetheless, there is a growing body of evidence demonstrating that these factors moderate children’s access to, use of, and effects from media. For instance, lower parent education, lower household income, and racial/ethnic minority status are associated with higher media use (Anand and Krosnick, 2005; Calvert et al., 2005; Wartella et al., 2014; Goh et al., 2016; Przybylski and Weinstein, 2017; Rideout, 2017). Context is also associated with specific media practices. For example, the extent to which parents coview or discuss TV content with children differs by race and ethnicity (Lauricella et al., 2017). Parents’ coviewing and active mediation in turn relate to how children comprehend, respond to, and learn from media (Valkenburg et al., 1999; Rasmussen et al., 2016; Piotrowski, 2017).

Media effects might be best understood through a family system lens. For instance, parent media use and child media use are correlated: higher parental media usage is associated with higher media usage by their young children (St.Peters et al., 1991; Bleakley et al., 2013; Connell et al., 2015; Nikken and Schols, 2015; Goh et al., 2016; Pempek and McDaniel, 2016; Anderson and Hanson, 2017; Lauricella et al., 2017). Parents’ own media use not only predicts their children’s media use, but it may also have an indirect effect on children via technoference (i.e., reduction in the quality of parent–child interactions when parents are engaged in their own media use) (McDaniel and Radesky, 2018). Parents are less actively engaged in their children’s play in the presence of adult-directed television (versus no television), resulting in lower levels of play (Kirkorian et al., 2019). Furthermore, parents’ use of mobile media during shared activities is associated with lower-quality interactions (Radesky et al., 2014, 2015a,b) and reduced learning (Reed et al., 2017).

While relatively few studies consider the impact of either content or context during early childhood, even fewer studies have investigated the interaction between these factors. This is illustrated in a 2017 systematic review of research on screen time and cognitive outcomes (Kostyrka-Allchorne et al., 2017), which included 39 cross-sectional and longitudinal studies of screen time during early childhood (0–5 years). After reexamining the articles in the review, we confirmed that two-thirds (62%) of these studies were based on a global estimate of screen time. Fewer than half (44%) considered content in any way. Among those that did consider content, 24% only distinguished between adult- and child-directed content, without measuring variations within child-directed programs (e.g., educational vs. entertainment).

Even fewer studies included in Kostyrka-Allchorne et al. (2017) systematic review considered the context of media use. For instance, we found that while 62% of the studies collected some type of data on parent–child interaction (e.g., parental warmth, parenting style, emotional, and cognitive stimulation), only 10% of the studies considered parent–child interaction as a potential *moderator* of media effects. Similarly, while nearly all of the studies (95%) reported data on one or more parent/child demographic characteristics (e.g., household income, parent education, child race, child ethnicity), only 10% considered these characteristics as potential moderators of media effects.

None of the studies reviewed by Kostyrka-Allchorne et al. (2017) examined interaction effects between media content and context. Although few studies consider such interactions (for exceptions during adolescence, see Linder and Werner, 2012; Fikkers et al., 2017), there is some evidence that individual children may be more or less susceptible to certain content effects–for good or ill–depending on individual- and family-level characteristics (Valkenburg and Peter, 2013). For example, there are fewer associations between media content and outcomes for children living in high-income homes; conversely, for children growing up in low-income homes, educational television is associated with better concurrent executive functioning, whereas background television predicts worse concurrent executive functioning (Wright et al., 2001; Linebarger et al., 2014). In lower-resourced families, educational media (e.g., television, apps, e-books) may be providing cognitive stimulation to children, which may have less impact in higher-resourced families (Linebarger et al., 2014).

In summary, it is critical to examine not only the quantity of media consumed, but also the content and context of early childhood media exposure (Barr and Linebarger, 2017). More precise measurement of the family ecology of early media exposure is needed in order to predict the long-term effects of media exposure on child outcomes. There is currently no standardized, systematic, scalable, and cost-effective measurement tool that comprehensively and accurately captures child and household media exposure, as well as the social context surrounding exposure during the first 5 years of life. The lack of such a tool represents a critical barrier for researchers who aim to describe child and family media use, identify characteristics associated with media use, evaluate associations between media use and concurrent behavior, and assess long-term developmental outcomes associated with early media use–for good or ill.

## Developing a Comprehensive Assessment of Family Exposure

### A Synergistic Science Approach

**FIGURE 1:**
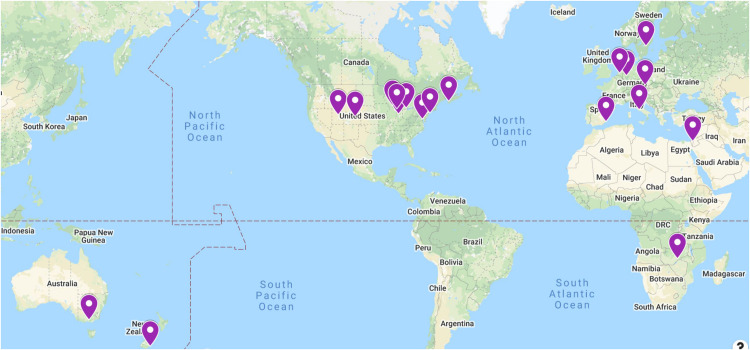
CAFE Consortium sites around the globe. Data collection is ongoing or planned at each site.

The Comprehensive Assessment of Family Media Exposure (CAFE) Consortium is an international group of cross-disciplinary collaborative researchers (Figure 1) formed in 2015 based on a shared interest in improving the quality of media measurement tools. So far, data have been collected at five sites across the United States, as well as in Canada, Germany, the Czech Republic, Sweden, Italy, the Netherlands, Australia, New Zealand, and Zambia. Data analysis and integration across these sites are ongoing. Taking a synergistic science approach, the CAFE Consortium developed a comprehensive assessment of family media exposure that included parental report of household usage patterns, attitudes, and practices, accompanied by detailed online time-use diaries and mobile device data collected via passive-sensing applications. Such a multipronged, multidisciplinary approach has been taken in the physical sciences to facilitate data integration and comparison across different sources and collection methods (Yip, 2003) and has recently extended to the field of developmental science (Gilmore and Adolph, 2017).

The purpose of the current article is to describe current challenges in measuring media use and introduce the state-of-the-art CAFE tools to demonstrate the feasibility of a synergistic data collection and analysis approach. The flagship journal of the Cognitive Development Society described tools in the field of developmental science using a tools-of-the-trade approach (e.g., Oakes, 2010). We have adopted a similar approach describing four primary challenges in the field and potential solutions that are offered by different CAFE tools. As part of this approach, we describe the development of each of the CAFE tools and consider their strengths and limitations. We also present illustrative preliminary data from our ongoing study for the purpose of demonstrating the utility of different CAFE measures for capturing different family media use constructs. Our synergistic approach will allow us to test the reliability, validity, and generalizability of these measures in future reports.

### The Ongoing CAFE Study

In order to provide preliminary data to illustrate how each of the tools can address a particular measurement challenge, we collected and integrated data from four sites across the United States. For context, we first summarize the protocol for an ongoing study. The rest of the article uses preliminary data from this study to illustrate key challenges to early media exposure assessment and how each of the CAFE tools can independently and collectively address these challenges. In this perspective piece, the preliminary data serve to illustrate key concepts. Validity and reliability testing of these measures is ongoing and will be published in a future report.

As of November 2019, data from 1074 participants were uploaded to a dashboard created and hosted by OpenLattice, Inc. These data were collected at the University of Wisconsin, University of Michigan, Brigham Young University, and Georgetown University. Each site had an independent institutional review board review. Participants provided informed consent to share data with the CAFE Consortium through the OpenLattice dashboard. Data were then subsetted to include only those families with children who were 0 to 72 months old and who responded correctly to at least 50% of quality assurance questions, resulting in *n* = 914 parents. Child participants were 431 girls (47% of sample, mean_age_ = 30.9 months, SD_age_ = 13.5 months) and 483 boys (53% of sample, mean_age_ = 29.97 months, SD_age_ = 12.8 months) between 0 and 72 months of age. Participants were drawn from a range of socioeconomic and educational backgrounds, although a majority had at least a 4-year degree: Respondents reported a high school education or less (*n* = 100, 11%), some college or an associate’s degree (*n* = 248, 27%), a bachelor’s degree (*n* = 272, 30%), and a master’s or doctoral degree (*n* = 294, 32%).

Participants completed one or more of the CAFE tools, described in detail later. Most participants were asked to complete an online questionnaire and an online time-use diary; these tools were developed first. Additionally, participants at some sites were asked to install a passive sensing app on their mobile device to track mobile usage. The app was developed for the Android operating system, so only those families with Android devices were able to utilize the app. Elsewhere we reported data from the CAFE passive-sensing app for Android and similar data collected from iOS devices, revealing few systematic differences between device use in Android and iOS users (Radesky et al., 2020). Most families (*n* = 624, 68%) provided data for the questionnaire and diary but not the passive-sensing app. Other families provided data for the questionnaire and passive-sensing app but not the diary (*n* = 27, 3%) and some the questionnaire only (*n* = 184, 20%). The remaining families (*n* = 79, 9%) provided data using all three tools.

## Challenges to Measurement and Best Practices

The primary goals of this perspective article are to describe current challenges in measuring media use and illustrate the feasibility of a synergistic data collection and analysis approach using preliminary data from the CAFE tools. We posit that many studies purporting to examine the relation between early media exposure and developmental outcomes fall short of achieving that goal because almost all ignore the content and context of that early media exposure, focusing predominantly on the total estimated amount of exposure to media. When researchers have examined the relationships between media content and context, they have reported much more nuanced and actionable findings (for review, see Barr and Linebarger, 2017). The rest of this perspective piece highlights four current challenges and how the CAFE Consortium has improved upon existing approaches by developing a comprehensive questionnaire that covers parental attitudes and new digital media (e.g., video chat and smart speakers) (challenge 1), an online time-use diary that emphasizes context (challenge 2), and a passive-sensing app that accurately tracks short bursts of mobile devise use (challenge 3). By combining information across these data streams, we examine how each of these components contributes to the overall household media ecology and index aspects of the content and context of early media exposure (challenge 4).

### Challenge 1: How Can Researchers Measure Attitudes and Practices?

In order to establish the context of media usage, it is important to assess not only the media environment (e.g., how many devices are owned), but also instrumental uses of media for the parent toward the child (e.g., calming or educating the child, occupying the child during travel), attitudes toward media (e.g., concerns about media effects), and household media practices (e.g., coviewing, location of devices, parental digital work demands). Finally, a number of demographic factors have been associated with media usage patterns. A parent-report survey is the method best suited to assess demographics, the general media environment, and parents’ attitudes and practices. We therefore developed a comprehensive survey, the Media Assessment Questionnaire (MAQ), to capture parent attitudes and behaviors around media.

#### Questionnaire Description

The 74-item questionnaire covers 10 topics, including household composition and demographics, parent mediation of media use, parent attitudes toward media use, and access to and regularity of use of different devices frequently found in the modern household. The approximate time to complete the entire questionnaire is 20–30 min. The questions were derived from a number of existing surveys (e.g., Lapierre et al., 2012; Rideout, 2017) and were updated to reflect current technologies and research on the content and context of early media exposure. For example, we updated Lapierre et al. (2012) representational survey of early media exposure to include newer devices (e.g., smart speakers, e-books, DVRs, tablet computers), content delivery mechanisms (e.g., streaming content), and newer technology-based activities (e.g., video chat). Quality check questions were embedded in the MAQ to ensure that participants were not responding randomly to questions. The illustrations in this perspective article are based on parents who were accurate on 50% or more of the quality check questions.

**FIGURE 2:**
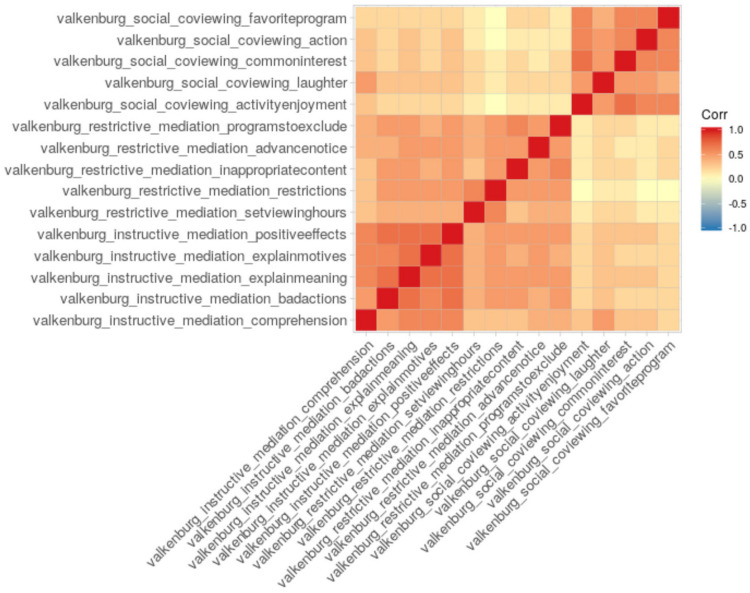
A correlogram depicting parents’ responses to the Valkenburg et al. (1999) mediation scale.

We also included established measures of parent media use, behaviors, and attitudes, such as Valkenburg et al. (1999) parent mediation scale. We use the data collected with the established Valkenburg scale to demonstrate our dashboard correlogram function using a measure with an established factor structure (Figure 2). In our preliminary data, we see evidence of a similar factor structure, particularly what Valkenburg et al. (1999) called *instructive* and *social* viewing patterns. With sufficiently large samples, such patterns could be analyzed for coherence across strata, such as family characteristics or study sites.

In future reports, we can use this utility to visualize and test factor structure for the newly developed MAQ. For example, we will visualize factor structure in questions about parental technoference (i.e., extent to which technology is seen to disrupt parents’ day-to-day activities) and about parents’ media-related concerns based on questions used in prior studies (Radesky et al., 2015b). The MAQ can easily be extended to include other questions or scales of interest to individual researchers. For instance, some of our investigators have included standardized parenting stress (Abidin, 1995), sleep (Sadeh, 2004), and language measures (Fenson et al., 2000) at the end of the MAQ.

#### Meeting the Challenge

A comprehensive questionnaire can best capture demographics, attitudes, and practices–factors known to be associated with household media usage patterns.

### Challenge 2: How Can Researchers Capture the Content and Context of Media Usage?

Most prior media exposure research focuses on global estimates of a child’s total time spent (Vandewater and Lee, 2009), often ignoring time of day, frequency of use, content, and context. This is true despite robust evidence that both content (Fisch, 2004; Anderson and Kirkorian, 2015) and context (Zack and Barr, 2016; Pempek and Lauricella, 2017) are critical moderators of media effects on learning, behavior, and development. Unlike global estimates, time-use diaries account for every moment in a particular day. Research demonstrates that time-use diaries produce more accurate estimates of actual media use than do global estimates of average media use in a “typical day” (Anderson et al., 1985). Moreover, diaries can be used to index the content and context of media usage within the daily activities of the child and family. Therefore, we developed the CAFE Time-Use Diary (TUD) to more accurately capture not only the amount, but also the content and context of media use.

#### Diary Description

**FIGURE 3:**
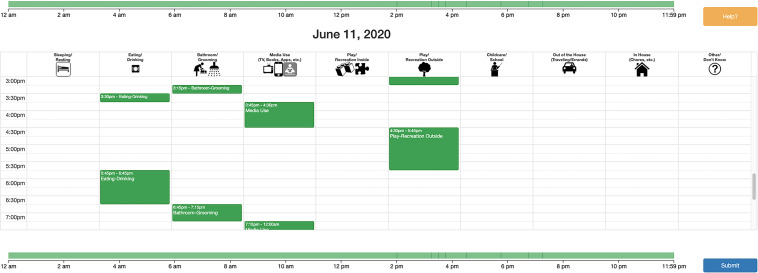
Illustration of a completed time-use diary. A help button contains instructional videos describing how to complete the diary, how to delete an activity, and how to know that the diary is complete. Parents click-and-drag to fill 15-min time blocks of child activities across the day. After creating a time block, they click on the time block to complete follow-up questions. The horizontal timeline at the top and the bottom of the diary indicates when each time block is complete by changing color from gray to green.

The CAFE TUD is a custom-designed, online 24-h time-use diary that details daily activities with follow-up questions about media content and context. The activities were derived from the Panel Study of Income Dynamics Child Supplement time use survey^1^ with the addition of a media-use category. There are 10 activity categories (sleep, media use, indoor play, outdoor play, travel, eating, grooming, childcare, household routines, other). Parents fill 15-min blocks of time indicating the target child’s primary activities throughout the day. See Figure 3 for a screenshot of a completed diary. After blocking out primary activities, parents answer follow-up questions that are customized to each primary activity category. For instance, parents answered follow-up questions about background media and parents’ own mobile device use for each block pertaining to sleep, eating, and play categories. For all blocks of time in the primary media-use category, parents answered follow-up questions about media content and coviewing patterns. Respondents take approximately 20 min to complete the TUD.

**FIGURE 4:**
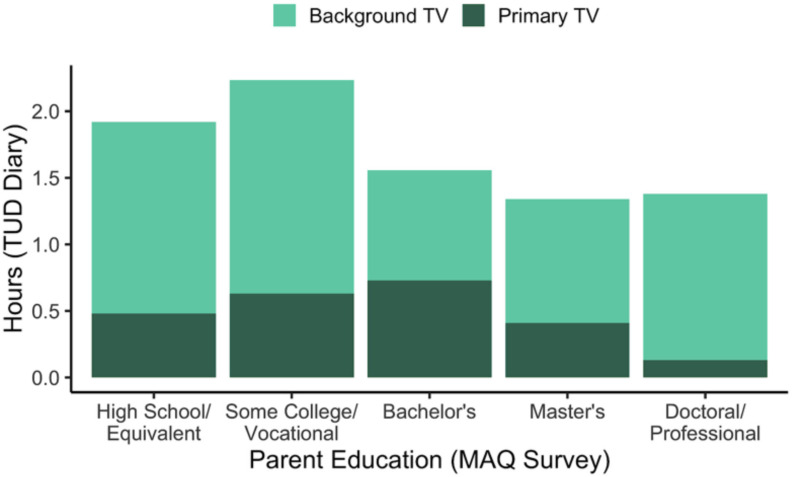
Mean parent-reported primary and background TV on the previous day (using the TUD) as a function of parent education (using the MAQ). Primary TV is reported by selecting media use as a primary activity on the time diary grid and indicating that the media type was TV during the follow-up questions about media content and context. Background TV is reported by selecting one of several other primary activities (e.g., sleeping, playing, eating) and indicating that TV was on in the background during the follow-up questions about context. This figure is based on a subset of participants with a TUD (*n* = 493, 70%) who provided their education level, reported at least 18 h (but not more than 26 h) on the diary and who opened the follow-up questions for at least 90% of the activity blocks in their TUD.

By capturing a high-resolution snapshot of one or more days in a child’s life, we can more accurately measure child media use in the context of other activities throughout the day. For instance, we can visualize primary media use and background media use reported in the TUD as a function of family characteristics reported in the MAQ. See Figure 4 for one illustration examining TV/video viewing as a function of parent education.

**FIGURE 5:**
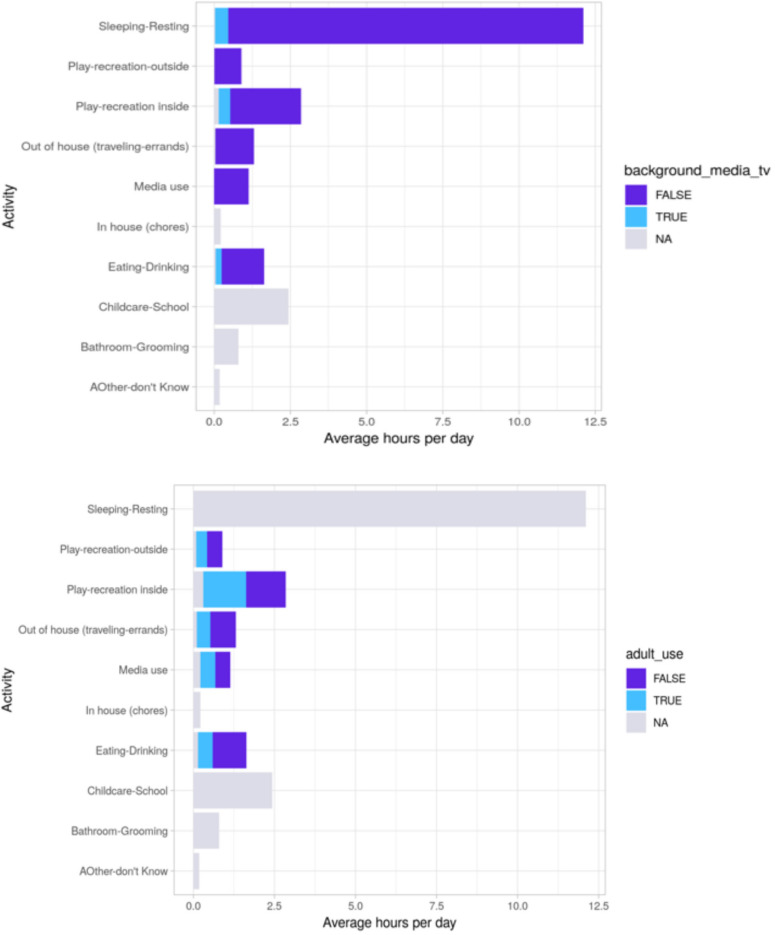
Illustration of the amount of time parents reported background TV **(top)** and parent mobile device use **(bottom)** were present (blue/true) or absent (purple/false) during each activity in the TUD. Background TV and parent mobile device use are reported during the follow-up questions about media context for some activities. Gray shading indicates that the follow-up questions did not ask about background TV or parent mobile device use for a particular category (if the entire bar is gray), or the parent did not answer the follow-up question for one or more blocks of time (bars with one small gray segment). This figure is based on a subset of participants with a TUD (*n* = 500, 71%) who reported at least 18 h (but not more than 26 h) on the diary and who opened the follow-up questions for at least 90% of the activity blocks in their TUD.

We can similarly capture the extent to which activities such as sleeping, eating, and playing are accompanied by background TV or parents’ own mobile device use. Both background media and parent media often disrupt ongoing child activity via a process described as *technoference* (McDaniel and Radesky, 2018). Figure 5 illustrates times when child activities may be disrupted by either background TV/video or by parent media usage that is unrelated to bedtime routines or mealtimes. Future analyses could examine the content of media use or variations in activities over the course of the day (e.g., the types of media used immediately before vs. during bedtime routines), whether differences in potential technoference are associated with sleep patterns, and many other questions regarding the context of media use.

#### Meeting the Challenge

As illustrated, time-use diaries are most useful as descriptions of larger blocks of time and providing context for media use, such as capturing co-occurring activities or determining who (if anyone) is with the child during each activity.

### Challenge 3: How Can Researchers Measure Short Bursts of Mobile Device Usage?

One of the goals of the CAFE Consortium is establishing reliable methods for measuring use of newer media (e.g., mobile devices) where exposure occurs in short bursts (Oulasvirta et al., 2005). Short bursts make retrospective assessments problematic (Burns and Anderson, 1993; Vandewater and Lee, 2009). Furthermore, the fact that even young children use handheld devices by themselves (Domoff et al., 2018) limits parents’ ability to correctly estimate their children’s usage. Moreover, mobile devices are multimodal computers, so measurement of app usage (e.g., video chat vs. YouTube vs. games), as well as the context (e.g., who is using the device), is needed in order to accurately characterize children’s media exposure. Although diaries have in the past been validated against direct observation of TV viewing with correlations ranging from 0.70–0.80 (Anderson et al., 1985), there are now many more household devices to track. Therefore, our converging method approach includes mobile device passive sensing.

Mobile device sensing is a methodology that harnesses data that mobile devices already collect, such as location, call logs, app usage logs, or battery usage–in order to study user behavior. User experience researchers have used this method for over 10 years to optimize smartphone design, but it has rarely been utilized as an objective measure of mobile device use (Hiniker et al., 2016; Elhai et al., 2018). The CAFE Consortium developed a mobile device sensing app (*Chronicle*) for Android devices in partnership with OpenLattice, Inc. This app generates accurate data on parent or child mobile media usage.

#### Description of *Chronicle* Mobile Device Sensing App

**FIGURE 6:**
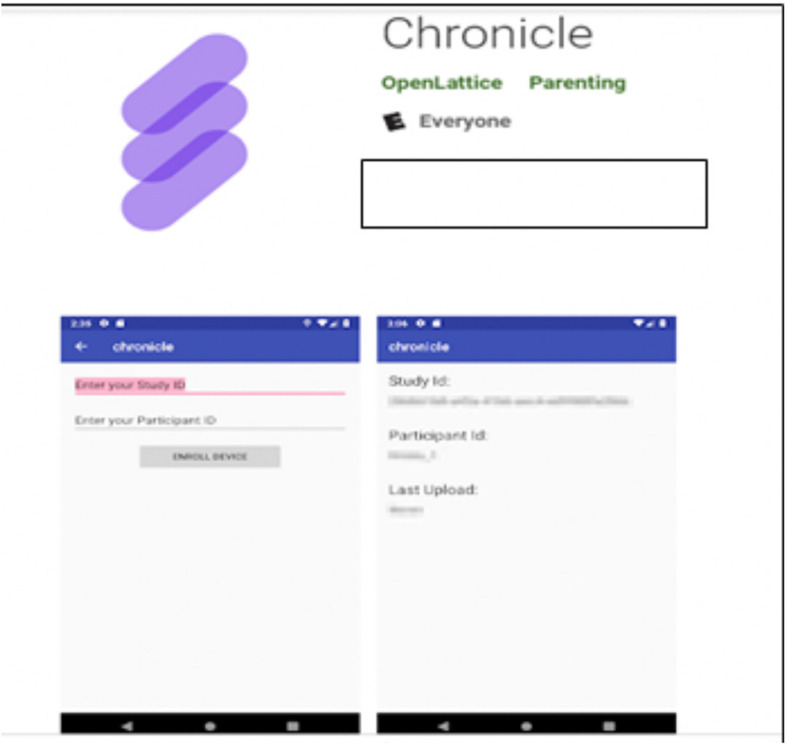
The interface that parents receive on downloading the *Chronicle* application from the Google Play Store. Participants receive a unique ID number.

The *Chronicle* app was custom-developed by OpenLattice, Inc., for Android devices (Figure 6). The *Chronicle* app and associated findings are described in more detail elsewhere (see Radesky et al., 2020). The *Chronicle* mobile device sensing app tracks the duration, frequency, time of day, general app type (e.g., email, phone, social media, educational), and app status (foreground vs. background, screen on vs. screen off) by querying the Google API every 15 min. Accessing the Google API for app usage statistics reduces computation and storage demands for participants. In addition, the Google API is used by millions of vendors and is closely monitored by Google for security, reliability, and accuracy. That is, participants give specific permission to allow researchers to access data that are already being collected by Google for the duration of the study. A limitation of this methodology is that only Android phone users currently are able to complete this study component. Efforts to expand to other operating systems are ongoing. As a stopgap, some CAFE Consortium investigators have used other methods such as embedded apps (e.g., *ScreenTime* for iOS) or other third-party apps (e.g., *Moment* for iOS). However, data collected by apps other than *Chronicle* are less detailed with respect to content and time intervals.

**FIGURE 7:**
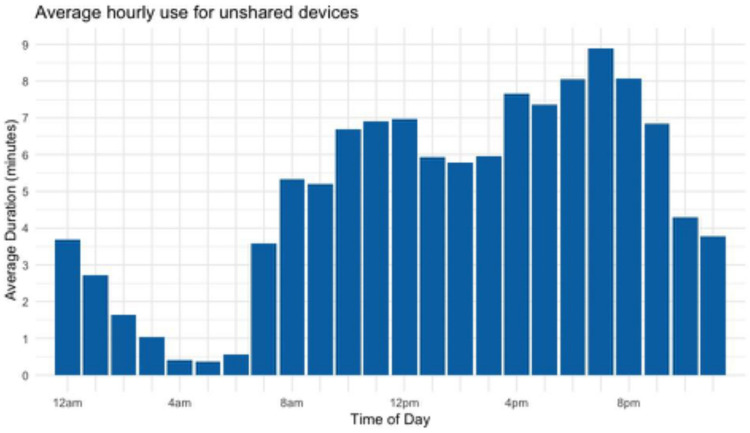
Average hourly usage by time of day, aggregated over all children with their own individual tablets as tracked by *Chronicle*. Generated from data collected in Michigan from August 2018 to May 2019. *n* = 37, aged 36–60 months.

Figure 7 illustrates some detailed information that can be obtained from *Chronicle* to address questions of usage across the day. It is possible to see daily fluctuations in usage of different types of content. For example, and perhaps not surprisingly, activity by children on their tablets peaks in the evening and decreases during the night, but some children are still actively using the tablet at that time. Additional reliability and validity testing to evaluate the app is ongoing.

#### Meeting the Challenge

Mobile device sampling is a promising method to track short spurts of mobile device usage with high temporal resolution.

### Challenge 4: No Single Tool Captures the Ecology of Household Media Usage

The solution to this challenge is to use a comprehensive assessment of family media exposure using converging methods and data integration across the three CAFE tools (MAQ, TUD, and *Chronicle*). Taking a synergistic approach, the CAFE Consortium is actively recruiting participants to complete the CAFE tools as part of their ongoing research programs. We developed a protocol for de-identifying and sharing data across sites for collation purposes to maintain confidentiality and maximize data sharing.

This suite of tools allows us to leverage the strengths of each methodology to provide the best metrics for parental attitudes via survey methodology (Barr and Linebarger, 2017; Valkenburg et al., 1999), capture the content and context of media use alongside all daily activities via online time use methods (Vandewater and Lee, 2009), and reduce participant bias in recalling small bursts of mobile device usage via passive sensing technology to automatically record digital media usage (Goedhart et al., 2015). This maximizes the pros of each measurement approach while offsetting the cons. For example, media usage does not occur in a vacuum. Continuous, intensive measurement of media use will be most informative when it is integrated with data about important drivers of media use such as participant mood (Bayer et al., 2016), cues (Bayer and Campbell, 2012), behaviors (Fedele et al., 2019), social interactions (Radesky et al., 2016), or other environmental or contextual variables. We posit that mobile device sensing is much more accurate for short bursts of time on mobile devices. Parental attitudes, however, influence how media are used (e.g., Valkenburg et al., 1999). These metrics can be accessed via standardized surveys. Meanwhile, the context of usage in larger chunks of time including who is present can be best captured by the time-use diary. Combining passive sensing of mobile media usage via *Chronicle* with the TUD and MAQ data allows the context of the media usage to be established. *Chronicle* and TUD can also capture aspects of media content (e.g., app or program titles).

#### Description of the CAFE-OpenLattice Dashboard

In order to integrate data streams from each of the CAFE tools, we built and tested a dashboard to accommodate use and storage by multiple study teams. We built an automated data pipeline for cleaning and visualizing data, including a customizable dashboard to facilitate standardized summary variable creation and reduction within the OpenLattice platform.

To maximize future utilization, the dashboard was developed in *R* (R Core Team, 2013), a widely used and freely available data analysis language for social sciences and available for collaboration on the code sharing platform GitHub. The dashboard has been built with consideration of international security and privacy regulations including Health Insurance Portability and Accountability Act and General Data Protection Regulation. Specifically, the dashboard requires a secure data login by each researcher. Each team deidentifies data before integrating into the platform. For example, each participant has a code number, and no names, dates of birth, or IP information about any participant is stored. The code numbers are recoded within the platform for added protection. Each research team can view tables of their own individual data to ensure accurate data upload and data integrity, but researchers cannot see individual data from other investigators (only aggregated data).

**FIGURE 8:**
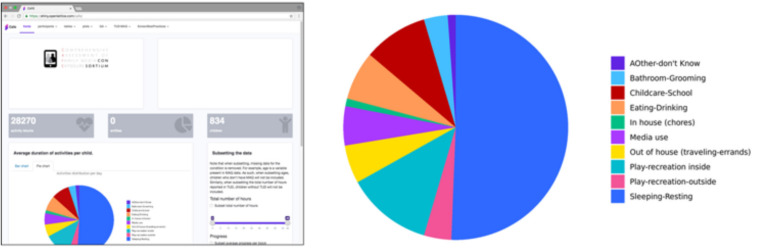
Screenshot from the dashboard landing page (left) that provides data visualization of the time use activities (expanded on the right), the number of participants, and options for subsetting the data for further visualization and analysis.

At the time of this writing, the platform integrates data for TUD and MAQ. See Figure 8 for a screenshot of the dashboard landing page. We are in the process of creating such a dashboard for the *Chronicle* app as well. Ultimately, the dashboard will allow for visualization and analysis of data across all three CAFE tools: MAQ (survey), TUD (diary), and *Chronicle* (mobile device sensing app).

**FIGURE 9:**
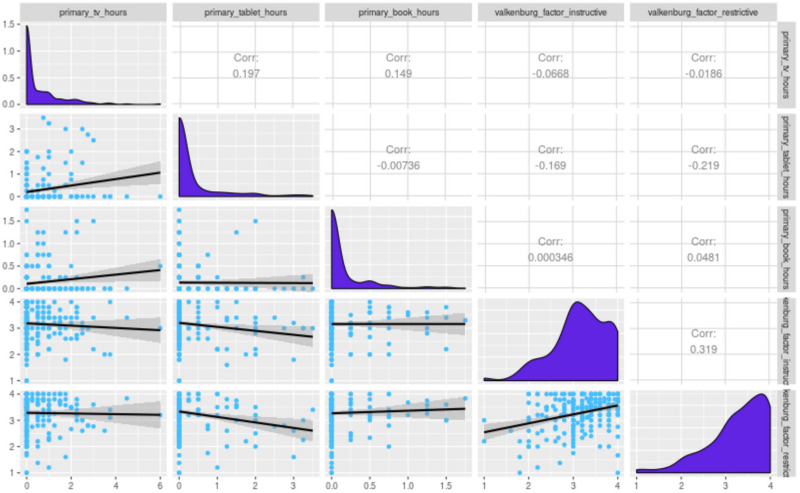
Distributions, scatterplots, and correlations between the primary TV hours, tablet hours, and book hours from the TUD and the Valkenburg instructive and restrictive scales from the MAQ. Along the diagonal are distributions of each variable. Above the diagonal are Pearson correlation coefficients. Below the diagonal are scatterplots between the variables. Initial visual inspection of the data suggests associations between tablet use and parental mediation may exist, which can be further tested in the dashboard. This figure is based on a subset of participants with a TUD (*n* = 231) who were between 30 and 72 months, reported at least 18 h (but not more than 26 h) on the diary, and opened the follow-up questions for at least 90% of the activity blocks in their TUD.

Using the current dashboard, we can examine associations between detailed reports of media use (as reported in the TUD) and a wide range of parent-reported household characteristics, child behavior, and outcomes (as reported in the MAQ). Preset scripts in the dashboard allow investigators to quickly and easily visualize distributions and associations. See Figure 9 for an illustration. In Figure 9, we show the distributions of and correlations between TV, tablet, and book use as the child’s primary activity (as opposed to being on in the background) from the TUD and the Valkenburg instructive and restrictive scales from the MAQ. Primary TV hours are associated with primary tablet and book hours. Figure 9 illustrates how the data distributions and associations between different variables are visualized.

**FIGURE 10:**
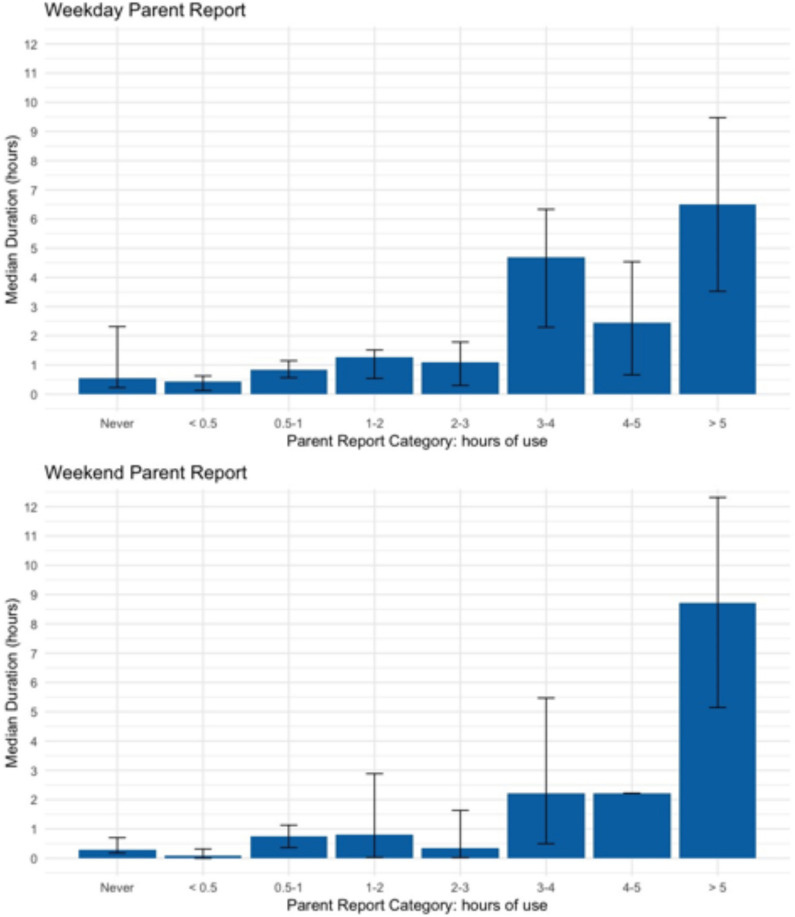
Comparison of parent-reported child mobile device use category from the MAQ to median daily usage calculated from *Chronicle* on weekdays (top) and weekends (bottom). This figure is based on a small sample collected in Michigan (*n* = 37 parents with a child 36–60 months old).

Given concerns about the accuracy of self-report estimates of small bursts of mobile activity, we examined the relation between self-reported mobile usage time [grouped by time estimates (from the MAQ)] and the time tracked by the *Chronicle* app in a group of 37 participants. These data collected with the MAQ and *Chronicle* illustrate that many parents were inaccurate (either overreporting or underreporting) when self-reporting their mobile device usage on the MAQ. Approximately one in three parents (31%) accurately reported mobile device use during weekdays, and only one in four (24%) accurately reported mobile device use during the weekend (Table 1). Figure 10 illustrates the concordance between *Chronicle* and parent report. For example, when parents self-reported 2- to 3-h usage on either the weekday or weekend using MAQ, *Chronicle* recorded 30 min less on the weekend and 1 h less during the weekday overall. In general, parents tended to either underreport or overreport on both weekdays and weekends. *Chronicle* data collection based on the Google API has been tested for accuracy against usage logs. In addition, comparison to parent reports suggests that, as in prior studies, self-report and recollection of cell phone usage are likely to be poor.

**TABLE 1:** Proportion of parents who were underreporting, accurate, or overreporting their preschool-aged child’s mobile device use, compared to *Chronicle* output, as a function of weekend or weekday estimates.

	% Underreport	% Accurate	% Overreport
Weekday	37.14%	31.43%	31.43%
Weekend	30.30%	24.24%	45.45%

We calculated Kendall’s tau-b rank correlation coefficient (*p* < 0.05) between the category of parent-reported mobile device use on the MAQ and the *Chronicle* estimate. These correlations were relatively low for weekdays, τ(35) = 0.41, *p* < 0.001, and not significant for weekend days, τ(33) = 0.20, *p* < 0.11. Conversely, the correlation between the two self-reported estimates (weekday and weekend day) was high, τ(35) = 0.81, *p* < 0.0001, suggesting that parent report is consistent within individuals but less consistent with more objective metrics. This finding illustrates that parent-reported mobile device use may not be a reliable measure of actual mobile device use. Passive mobile device sensing is a more reliable and accurate way to measure child mobile phone usage (see also Radesky et al., 2020).

**FIGURE 11:**
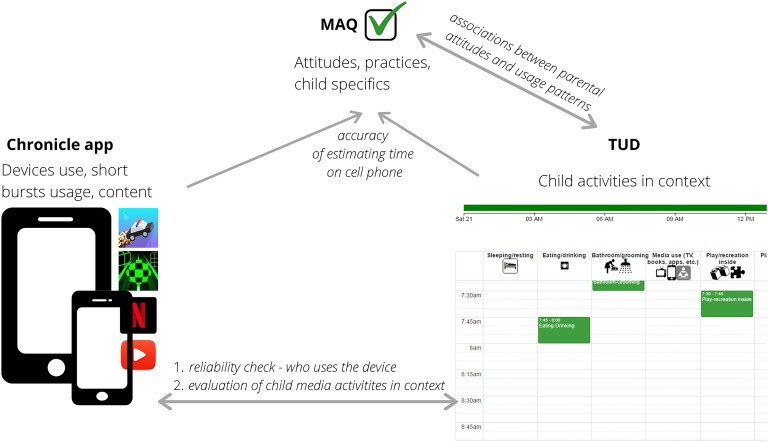
Conceptual overview linking the MAQ, TUD, and *Chronicle* to support reliability and validity testing. This work is ongoing.

The examples presented here illustrate the utility of combining multiple methods to better capture the family media ecology. Ultimately, we will be able to test reliability and validity by testing for consistency across all three tools. Figure 11 provides a conceptual overview linking the MAQ, TUD, and *Chronicle* to support future reliability and validity testing.

#### Meeting the Challenge

The combination of methods provides a more comprehensive assessment of the family media ecology, creating opportunities for improved validity and reliability testing.

## Discussion

We have successfully applied a synergistic approach to developing the CAFE tool. There are a number of advantages of the synergistic scientific approach taken by the CAFE Consortium. Consortium members have complementary expertise in different developmental domains (e.g., memory, language, sleep, pediatrics). Each research group contributed to the design of the CAFE tool, collected data using the tool, and also designed and collected data with other specific research questions in mind. That is, all researchers have a shared interest in the measurement of household media ecology, but all have independent research programs. For example, research groups are pursuing links to attachment, mental health, language development, book reading, and language and sleep patterns. Experimental studies are evaluating technoference and physiological responses to media exposure. All researchers collect and collate the data from the CAFE tools in the dashboard. Meanwhile, each researcher can use his/her own data as a metric within individual designs. From the open-science framework perspective, however, it is useful for multiple groups to utilize the same tool and to share data in order to replicate across multiple sites. Data collection time is reduced and optimized. From a data analysis perspective, analytics can be optimized across the datasets for data visualization and analysis. This allows researchers to then develop and test more complex questions based on a larger and more diverse sample. Finally, it is feasible to do cross-site comparisons to assess whether variables that differ across sites (e.g., culture, population density, language) can be directly compared because the same metrics have been developed by and utilized across sites. Thus far, materials have been translated into Spanish, Czech, Swedish, German, and Italian by Consortium members, and data have been collected in the Czech Republic, Italy, Germany, and Sweden, with plans to expand to other languages as needs and resources arise.

The content and context of early media exposure are likely to shape developmental trajectories and to be even more pronounced in the current media landscape than ever before (Barr and Linebarger, 2017). In this time of unprecedented technology expansion, researchers need better tools to track family media ecology and child responses to such exposure. The CAFE Consortium has taken a first step toward developing tools for the greater research community that can be utilized in longitudinal studies to examine how developmental trajectories of media exposure affect child outcomes. The OpenLattice dashboard provides an opportunity for researchers to rapidly integrate, visualize, and compare responses across a suite of complementary tools to establish best practices in measurement of family media ecology. As demonstrated here, each tool can meet different challenges. The MAQ can assess parental attitudes, practices, and household characteristics that influence the general household media environment. The TUD characterizes the broad duration of the child’s daily activities in the context of media patterns in the household, including follow-up questions about the content and context of media use. Finally, mobile device sampling via *Chronicle* provides a detailed assessment of the short bursts of mobile activity and different content accessed throughout the day. As illustrated here, the CAFE tools can be used to replicate investigations of factors that are likely to be associated with screen time within the family context, such as associations between education and daily duration of media use. Such a replication approach will allow us to further test the reliability and validity of the tools.

### Future Directions

The CAFE tools can be used to extend our knowledge of family media ecology, going beyond the default screen time estimates to test which combination of factors is likely to predict child outcomes. There are a number of exciting directions that the CAFE Consortium hopes to pursue.

#### Patterns Across Time of Day

Both TUD and *Chronicle* are time-stamped throughout the day, and we are currently integrating these time-stamped activities. In the future, this degree of time-stamped information will allow us to map blocks of time in different activities from the TUD (e.g., mealtime, play, hour before sleep) to blocks of usage by parents on their devices or children’s own tablets. For example, we can plot number of engagements and duration of engagements on mobile devices during outdoor playtime or mealtime.

#### New Data Streams and Analytics

Currently, we are building upon our existing dashboard to incorporate additional time-stamped objective measures. For example, different CAFE groups are currently collecting data using a number of wearable devices to track physiological responses, including heart rate variability, actigraphy, LENA audio recordings of the language environment, and ecological momentary assessment (EMA). Specifically, EMA participants receive additional contact during the mobile device sampling and TUD data collection periods to collect contextual data such as a panoramic photo. We would build upon approaches developed to examine adolescent media usage using MYME (e.g., Rich et al., 2015). New assessments also need to capture emerging technologies like video chat, virtual reality, and intelligent agents. The addition of these time-stamped methods would allow researchers to examine cascades of events that shape behavior over minutes, hours, or days–rather than asking for global estimates. Integrating mobile device sampling data with physiologic sensory data would allow us to identify physiologic stress, sleep patterns, or physical activity patterns crucial for understanding associations of media use with these health determinants.

#### Scalability and Sharing

We are streamlining the existing tools and integrating them within one interface to facilitate future scalability. We are actively testing reliability and validity. Concurrently, we are expanding our data analytics to address these complex contextual research questions. Simultaneously, we are exploring whether we can use our integrated data to develop a short form of household media usage. A short form might capture key features of the content and context of media exposure utilizing specific aspects of each of the three metrics with high validity, reliability, and relevance to important outcomes. These short-form CAFE tools could then be more easily incorporated into large-scale longitudinal studies. A short form will also be valuable for responding to time-sensitive research needs. For instance, some Consortium members have used CAFE tools to capture family media ecology following a natural disaster. Similarly, the COVID-19 pandemic emerged as we wrote this perspective article, dramatically changing the media landscape for millions of young families and creating a critical need to understand how media may help or hinder as families cope. Ultimately, once the Consortium has completed the dashboard and analytics phases of the CAFE tool development, we will share and scale the tool for broader usage.

#### Associations With Cognitive and Behavioral Assessments

We are currently examining associations between media exposure and daily activities with other standardized measures. For instance, many sites added a standard sleep questionnaire (Sadeh, 2004), parenting stress questionnaire (Abidin, 1995), and infant language measures (Fenson et al., 2000). We will therefore examine associations between the content and context of early media exposure with parenting stress, child sleep duration and quality, child language, and changes to routines such as bedtime reading.

#### Comparing Attitudes and Activities

Optimizing the three tools, we aim to examine patterns of short bursts of mobile device activity (*Chronicle*) with self-reports of attitudes toward mobile usage (MAQ) and selected child activities (e.g., play and mealtimes from the TUD). We could then test predictions about relations between attitudes, activities, and usage patterns.

#### Cross-Cultural Comparisons

We are now integrating data that have been collected at sites outside of the United States to assess global similarity in the adoption and use of technology in the household. We will be able to examine cross-cultural comparisons of attitudes and availability of media in different countries, as well how specific policy (e.g., parental leave policies, income inequity, privacy regulations) may be associated with household media practices.

As shown by the challenges outlined in the current article, the standard approach to media assessment is insufficient. As illustrated by the comprehensive, converging, and complementary nature of the CAFE tools, parent global estimates can be inaccurate or provide an incomplete picture of the context of media exposure. When comparing mobile device sampling to self-report, a majority of parents either overestimated or underestimated their actual device use. More accurate assessment is needed not only for researchers, but also for healthcare, home visitors, and childcare providers in order to develop guidelines for healthy media diets that are based on realistic usage patterns, highlighting both problematic and effective practices. Better feedback can then be provided to parents. Obvious health domains that require further investigation are in the areas of parents’ usage, parental stress, child obesity, child emotion regulation, and cognitive outcomes. It is critical that future studies include diverse populations, across race, ethnicity, and income. It is equally important that measures of family media ecology are easy for participants to use. The finalized CAFE tools could be utilized globally to examine child health and welfare, where there is a critical need to incorporate more precise measures of media exposure to go beyond screen time.

## Data Availability Statement

The datasets generated for this study will not be made publicly available. We have built a data sharing platform and will be able to share secure access to the dataset once the platform has been completely built and data integrated from Consortium members. Requests to access the datasets should be addressed to the corresponding author.

## Ethics Statement

The studies involving human participants were reviewed and approved by the University of Wisconsin–Madison, Georgetown University, University of Michigan Medical School, and Brigham Young University. Written informed consent to participate in this study was provided by the participants’ legal guardian/next of kin.

## Author Contributions

All authors have contributed substantially and in a meaningful way to the manuscript. All authors are original members of the CAFE Consortium that conceptualized the CAFE suite of tools. Data has been collected by all authors except the data scientists, ME and JD who built the OpenLattice dashboard where the data were integrated. The suite was translated into Swedish, German, Italian and Czech by Sundqvist, Konrad, Bellagamba, and Slussareff respectively before implementing the data collection in each country. RB, JR, SC, and DN wrote the grant and were awarded funding to build the database.

## Conflict of Interest

JD and ME were employed by company OpenLattice Inc. The remaining authors declare that the research was conducted in the absence of any commercial or financial relationships that could be construed as a potential conflict of interest.

## Footnotes

1 https://psidonline.isr.umich.edu/Guide/default.aspx
